# Antioxidant capacity of hydrolyzed porcine tissues

**DOI:** 10.1002/fsn3.106

**Published:** 2014-03-21

**Authors:** Trine D Damgaard, Jeanette A H Otte, Lene Meinert, Kirsten Jensen, René Lametsch

**Affiliations:** 1 Food chemistry, Department of Food science, University of CopenhagenRolighedsvej 30, Frederiksberg C, DK-1958, Denmark; 2 Danish Meat Research InstituteMaglegaardsvej 2, Roskilde, DK-4000, Denmark

**Keywords:** Antioxidant capacity, enzymatic hydrolysis, meat by-products, pig

## Abstract

The antioxidative capacity of seven different porcine tissue hydrolysates (colon, appendix, rectum, pancreas, heart, liver, and lung) were tested by four different assays, including iron chelation, 2,2′-azino-bis (3-ethylbenzothiazoline-6-sulfonic acid) (ABTS) radical scavenging, 2,2-Diphenyl-1-(2,4,6-trinitrophenyl) hydrazyl (DPPH) radical scavenging, and inhibition of lipid oxidation. All hydrolyzed tissues displayed antioxidant capacity in all four assays, with colon, liver, and appendix as the three most potent inhibitors of lipid oxidation (47, 29, and 27 mmol/L trolox equivalent antioxidant capacity [TEAC], respectively) and liver, colon, pancreas, and appendix as the four most potent iron chelators (92% ± 1.1, 79.3% ± 3.2, 77.1% ± 1.8, and 77% ± 2.3, respectively). Furthermore, colon and appendix showed good radical scavenging capacities with ABTS scavenging of 86.4% ± 2.1 and 84.4% ± 2.9 and DPPH scavenging of 17.6% ± 0.3 and 17.1% ± 0.2, respectively. Our results provide new knowledge about the antioxidant capacity of a variety of animal by-products, which can be transformed into antioxidant hydrolysates, thereby creating added value.

## Introduction

Oxidation is one of the major causes of deterioration in food products, leading to unfavorable changes in flavor, texture, and color. Oxidation impairs the nutritional quality of foods by spoilage of vitamins and essential fatty acids such as linoleic and linolenic acid (Kirk 1984). Moreover, research has shown that consumption of oxidized oil in feeds can lead to in vivo oxidative stress in chickens (Zhang et al. 2011). Therefore, it is crucial that both foods and the living body are protected from excessive oxidation. The addition of, or the preservation of, existing antioxidants is one way of achieving this.

The use of in vitro controlled enzymatic hydrolysis of food proteins is progressively gaining interest as a source of bioactive hydrolysates and/or peptides. By choosing specific enzymes, a parent protein can be hydrolyzed to yield a variety of different peptides with different activities. These activities include antioxidant (Guo et al. 2009); Liu et al. 2011), antimicrobial (Jang et al. 2008), anti-hypertensive (Correa et al. 2011), and anticancer (Kannan et al. 2011) activities, to name but a few. The meat industry produces large quantities of low-value by-products, and bioactive peptides liberated from such materials could potentially be used as beneficial ingredients in functional foods or as natural preservatives in food items. The use of synthetic antioxidants such as butylated hydroxyanisole (BHA) and butylated hydroxytoluene (BHT) is currently restricted in many countries, because they have shown potential as carcinogenic agents (Ito et al. 1986). These could advantageously be replaced by antioxidant peptides derived from hydrolyzed by-products and other muscle foods. Due to their origin in food with a long history of consumption by man, such hydrolysates are considered as natural ingredients/preservatives. Antioxidant peptides have so far been reported from chicken (Sun et al. 2012), fish (Li et al. 2012), bovine brisket protein (Di Bernardini et al. 2012), porcine hemoglobin (Liu et al. 2011; Alvarez et al. 2012), porcine skin gelatine (Li et al. 2007) and porcine myofibrillar protein (Saiga et al. 2003).

In this study, we examined the antioxidant capacity of hydrolysates obtained from seven different porcine tissues with a high variation in chemical composition using a number of assays, testing for different antioxidant mechanisms (iron chelation, 2,2′-azino-bis (3-ethylbenzothiazoline-6-sulfonic acid) [ABTS] radical scavenging, 2,2-Diphenyl-1-(2,4,6-trinitrophenyl) hydrazyl (DPPH) radical scavenging, and lipid oxidation in emulsion) in order to assess (1) the usefulness of these by-products as antioxidants, (2) differences between tissues and (3) the underlying mechanism of any potential antioxidant capacity.

## Methods and Materials

### Chemicals

Protamex and Alcalase L 2.4 FG were purchased from Novozymes (Bagsvaerd, Denmark). Sodium dihydrogen phosphate (NaH_2_PO_4_), disodium hydrogen phosphate (Na_2_HPO_4_), extran neutral MA02, (2,2′-azino-bis (3-ethylbenzothiazoline-6-sulphonic) acid [ABTS]), and potassium persulfate (K_2_S_2_O_8_) were purchased from Merck (Darmstadt, Germany). Iron (II) sulfate (FeSO_4_) and 3-(2-Pyridyl)-5,6-diphenyl-1,2,4-triazine-p,p′-disulfonic acid monosodium salt hydrate (Ferrozine) were purchased from Sigma-Aldrich (St. Louis, MO). 1,1-diphenyl-2-picrylhydrazyl (DPPH) and 6-hydroxy-2,5,7,8-tetramethylchroman-2-carboxylic acid (Trolox), sodium ascorbate, hydrochloric acid, methyl linoleate, and tween-20 were purchased from Sigma-Aldrich (Steinheim, Germany). Hemin was purchased from Acros Organics (Geel, Belgium). Ethanol (99.9%) was purchased from Chemethyl A/S (Køge, Denmark), and sodium hydroxide was purchased from Sigma-Aldrich (Renningen, Germany).

### Preparations of hydrolysates

Porcine colon, appendix, rectum, pancreas, heart, liver, and lung tissue were collected from a slaughterhouse and stored at 4°C until use. Organs from several animals were initially minced (hole size 3 mm), and ∼1 kg of each tissue mixed 1:1 with water (w/w) and then heated to 55°C in a water bath while being stirred. A 1:1 mixture of Protamex and Alcalase 2.4 L FG (Novozymes) was added to a final enzyme:substrate ratio of 1:1000 (w/w), and the reaction was allowed to proceed at 55°C for 2 h. Hydrolysis was stopped by heating the samples for 10 min at 95°C. Samples were centrifuged at 2000*g* in a Rotafix 32A Hettich (Tuttlingen, Germany) for 5 min in order to remove lipids and insoluble proteins. The clear aqueous phase was subsequently frozen at −20°C until use.

### DM determination

The dry matter (DM) content was determined with a moisture analyzer (Sartorius MA50, Goettingen, Germany) by measuring the samples moisture loss at 130°C until constant weight. Approximately 3 g per sample was used for the analysis.

### Inhibition of lipid oxidation in emulsions

The oxygen consumption rate was assayed according to the protocol of Hu and Skibsted (2002) with some modifications. Methyl linoleate was mixed with Tween 20 and thermostated (25°C) phosphate buffer (pH 6.8), and 20 *μ*L of antioxidant solution and 25 *μ*L 0.20 mmol/L hemin aqueous solution to initiate the oxidation. The total volume was 2.8 mL. As a positive blank, 20 *μ*L ethanol was used instead of antioxidant solution. The measurement of oxygen consumption was performed under water (25°C) with an oxygen microsensor (Micro-respiration System, Unisense, Aarhus, Denmark) and recorded at 10-sec intervals for 10 min. Oxygen consumption data were collected as a function of time, and the slope of the curve in the linear region was used to calculate the initial oxygen consumption rate [*V*(O_2_)]. Data were processed with MicOX software (Unisense, Aarhus, Denmark), and an oxygen consumption index (*I*_oxygen_) was calculated according to the following equation:


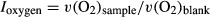


where *v*(O_2_)_sample_ is the initial oxygen consumption in the presence of the hydrolysate and *v*(O_2_)_blank_ is the initial oxygen consumption where ethanol has replaced the sample. The oxygen consumption of the samples was determined based on a Trolox standard curve (4, 2, 1 and mmol/L), with the index (*I*_oxygen_) as a linear function of the trolox concentrations, and expressed as Trolox equivalent antioxidant capacity (TEAC, mmol/L). All measurements were performed in duplicate and reported as the average value.

### Determination of iron chelation capacity

The iron-chelating capacity of the hydrolysates was investigated as the ability to inhibit the formation of a Fe^2+^- ferrozine complex, based on the protocol of Wu et al. (2007). All hydrolysate samples were filtered through a 0.45-*μ*m filter and diluted with distilled water to 5 mg/mL. Samples of 25 *μ*L were mixed with 100 *μ*L 75 *μ*mol/L FeSO_4_ and incubated for 10 min before adding 100 *μ*L of 500 *μ*mol/L ferrozine. The absorbance of the resulting mixtures was measured at 562 nm with the Multiskan EX microplate reader from Labsystems (Helsinki, Finland). The percentage of inhibition of the Fe^2+^- ferrozine complex formation was calculated by the following equation:


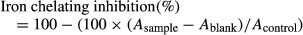


where *A*_sample_ is the absorbance of the Fe^2+^- ferrozine complex mixed with sample, *A*_control_ is the absorbance of the Fe^2+^- ferrozine complex mixed with water, and *A*_blank_ is the absorbance of sample and Fe^2+^ where ferrozine has been replaced with water. All measurements were performed in triplicate and reported as the average value.

### ABTS radical scavenging capacity

The radical scavenging capacity of the hydrolysates was assayed with an ABTS assay according to the protocol of Jensen et al. (2011), with some modifications. The ABTS radical solution (19.4 mmol/L ABTS and 6.7 mmol/L potassium persulfate) was diluted with 10 mmol/L phosphate buffer, pH 7.4 until A_405nm_ reached 0.7. All samples were filtered through a 0.45-*μ*m filter and diluted with distilled water to 50 *μ*g/mL. Samples of 50 *μ*L were subsequently mixed with 200 *μ*L ABTS radical solution, and the absorbance of the resulting mixtures was measured after 1 h at 405 nm with the Tecan Genios Plus microplate reader (Grödig, Austria). The scavenging capacity was calculated by the following equation:


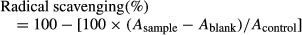


where *A*_sample_ is the absorbance of the ABTS mixed with sample, *A*_control_ is the absorbance of the ABTS mixed with water, and *A*_blank_ is the absorbance of sample mixed with water. All measurements were performed in triplicate and reported as the average value. Trolox (32 *μ*mol/L) was used as a reference.

### DPPH radical scavenging capacity

Measurement of the DPPH radical scavenging capacity was based on the work by Farvin et al. (2010). Briefly, 2 mL of 0.1 mmol/L DPPH in 20% ethanol was mixed with 2 mL sample containing 20 mg DM in 6.25% ethanol. After 30 min incubation, the absorbance was measured at 520 nm with a Lambda 2 UV/VIS spectrometer (Perkin Elmer, Ueberlingen, Germany). The percentage of radical scavenging capacity was calculated by the following equation:


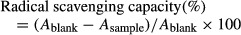


where *A*_sample_ is the absorbance of DPPH mixed with hydrolysate and *A*_blank_ is the absorbance of DPPH in which hydrolysate has been replaced with 6.25% ethanol. All measurements were performed in triplicate and reported as the average value. Trolox (0.25 mmol/L) was used as a reference.

### Statistical analysis

Data were expressed as means ± standard deviations, except for the results from the lipid oxygen inhibition, where the mean values are indicated with an estimate of inverse variance. Differences in the iron chelation, DPPH and ABTS radical scavenging capacities among the hydrolysates were analyzed by one-way analysis of variance (ANOVA) and Tukey's test (Microsoft Excel 2010). Differences in the oxygen consumption mean values among the hydrolysates were analyzed with 95% confidence intervals. Differences were considered statistically significant at *P* < 0.05.

## Results and Discussion

### DM content of hydrolysates

The DM content in the porcine tissue hydrolysates after 2 h of hydrolysis is shown in Table 1. As can be seen, the soluble yield varied from 5.9% to 13.8%, indicating differences in tissue types and enzyme catalytic site availability. Liver and pancreas gave the highest yields, which is in agreement with the higher protein content normally found in these tissues (21.39% and 18.56%, respectively). Heart and lung tissues contain less protein than liver and pancreas (17.27% and 14.8%, respectively) (cf. Table 2.1 Anderson 1988), which is also reflected by the lower DM contents in the resulting hydrolysates (Table 1). Finally, colon, appendix and rectum, which are all part of the large intestine, gave the lowest yields. This could be due to their lower protein content (12.6%; Gault and Lawrie 1980) and high amount of connective tissue compared to the rest of the tissues. According to the work by Gault and Lowrie, collagen constitutes 23% of the total protein content in the large intestine, whereas liver, heart, and pancreas contain only 3.65%, 7.54% (L. Meinert, unpubl.data) and 5.5% collagen, respectively (Hilling et al. 2009). Collagen is rich in glycine, proline, hydroxyproline, and alanine (Eastoe 1955), all of which possibly contribute to a lower solubility under the hydrolysis conditions in this study. Furthermore, the laminar structure of the intestinal tissue could present a structural hindrance of the enzyme accessibility, resulting in lower hydrolysis efficiency and hence a lower yield compared with the other tissues. Overall, the yield of the different hydrolysates fits quite well with the corresponding protein contents.

**Table 1:** Dry matter content% (w/w) of the hydrolysates

Liver	13.8
Pancreas	13.4
Lung	9.3
Heart	8.7
Rectum	7.9
Appendix	6.7
Colon	5.9

### Antioxidant capacity of hydrolysates

The antioxidant capacities of the porcine tissue hydrolysates assayed at comparable DM concentrations are shown in Table 2.

**Table 2:** Antioxidant capacity of the hydrolysates measured by iron chelation, ABTS and DPPH radical scavenging and inhibition of lipid oxidation

		Antioxidative capacity%^2^		
		
Sample	TEAC (mmol/L)^1^ Lipid oxidation inhibition^3^	Iron chelation^4^	ABTS^5^	DDPH^4^
Colon	47^a^ (CI 37–61)	79.3 ± 3.2^b^	86.4 ± 2.1^a^	17.6 ± 0.3^b^
Appendix	27^b^ (CI 22–36)	77.0 ± 2.3^b^	84.4 ± 2.9^ab^	17.1 ± 0.2^b^
Rectum	13^c^ (CI 9–18)	66.5 ± 3.3^c^	82.1 ± 3.8^ab^	12.1 ± 0.3^d^
Pancreas	19^bc^ (CI 10–30)	77.1 ± 1.8^b^	84.3 ± 3.4^ab^	13.4 ± 0.2^c^
Liver	29^ab^ (CI 22–38)	92.0 ± 1.1^a^	79.2 ± 4.2^ab^	9.9 ± 0.3^e^
Lung	22^b^ (CI 18–24)	38.0 ± 2.4^d^	87.9 ± 4.1^a^	9.7 ± 0.2^e^
Heart	14^c^ (CI 13–16)	20.8 ± 9.3^e^	76.5 ± 7.2^b^	25.4 ± 0.3^a^
Trolox			59.9 ± 7.8	13.9 ± 2.9

Values with different lowercase letters in the same column are significantly different at *P* < 0.05. ABTS, 2,2′-azino-bis (3-ethylbenzothiazoline-6-sulfonic acid); DPPH, 2,2-Diphenyl-1-(2,4,6-trinitrophenyl) hydrazyl; TEAC, trolox equivalent antioxidant capacity.

1 Values for inhibition of lipid oxidation are means with 95% confidence intervals (CI).

2 Values for iron chelation, ABTS, and DPPH radical scavenging are means ± standard deviations.

3 Inhibition of lipid oxidation was tested at 20 *μ*L hydrolysate converted to 100% DM.

4 Iron chelation and DPPH radical scavenging was tested at 5 mg/mL and trolox at 0.25 mmol/L.

5 ABTS radical scavenging was tested at 50 *μ*g/mL and trolox at 32 *μ*mol/L.

#### Inhibition of lipid oxidation in emulsions

The ability of the hydrolysates to inhibit lipid oxidation in a methyl linoleate system, revealed by a decreased oxygen consumption, is shown in the first column of Table 2. Colon displayed the highest inhibition of oxygen consumption, three times higher than that of heart and rectum.

#### Iron chelation capacity

Transition metals, such as iron and copper, can be categorized as pro-oxidants, as they can catalyze the formation of radical oxygen species and stimulate lipid oxidation (Stohs and Bagchi 1995; Skibsted 2010). Hence, compounds that chelate these metals are considered to have some antioxidant capacity. The hydrolysates were tested for their Fe^2+^ chelating ability at concentrations of 5 mg/mL. As seen in Table 2, the liver hydrolysate had the significantly highest capacity, followed by colon, pancreas, and appendix, all of which shared similar values. Lung and heart tissues, however, displayed much lower activities, representing weak chelating properties compared with the other hydrolysates. The values from liver, colon, pancreas, appendix, and rectum are comparable to the iron-chelating capacity reported for enzymatic hydrolysates of porcine hemoglobin (Chang et al. 2007), bovine brisket (Di Bernardini et al. 2012), and for tilapia fish protein hydrolysates (Foh et al. 2010), all assayed at 5 mg/mL. Furthermore, the values are higher than those of porcine plasma protein hydrolysates obtained from Alcalase hydrolysis for 0.5–5 h assayed at 40 mg/mL (Liu et al. 2010). This shows that these porcine by-products are transformed into potentially valuable antioxidant ingredients.

#### Radical scavenging capacity

Another mechanism by which peptides may exert antioxidant activity is by scavenging of radicals, which could otherwise initiate or propagate lipid oxidation (Skibsted 2010). The radical scavenging capacities were assessed with the lipid soluble DPPH radical as well as the water soluble ABTS radical.

##### ABTS radical scavenging capacity

The ABTS assay is based on the ability of an antioxidant to transfer electrons to, or donate hydrogen atoms to, a preformed ABTS radical cation, causing a change in color and a decrease in absorbance (Re et al. 1999). As seen in Table 2, the hydrolysates were tested for ABTS radical scavenging capacity at concentrations of 50 *μ*g/mL. The lung hydrolysate was the most efficient at ABTS scavenging but was not significantly different from the other hydrolysates, except for heart. Heart showed the lowest scavenging ability and was significantly different from lung and colon. The activity values were similar, ranging from 87.9% to 76.5% radical scavenging capacity. These values are quite similar to the radical scavenging activity of tilapia fish protein hydrolysate (66 *μ*g/mL), ranging from 88.13% to 94.23% obtained with different enzymes (Foh et al. 2010) and are higher than the values reported for hydrolysates of tannery fleshings (Balakrishnan et al. 2011) and fermented shrimp biowaste assayed at 50 *μ*g/mL (Sachindra and Bhaskar 2008), once again highlighting the value of the porcine tissues as potential substrates for antioxidant hydrolysates.

##### DPPH radical scavenging capacity

Like ABTS, DPPH is a radical which, upon scavenging by antioxidants, will change color, resulting in a decrease in absorption (Blois 1958). Table 2 presents the radical scavenging capacity of the hydrolysates at 5 mg/mL. In contrast to ABTS, the hydrolysate from heart showed the strongest DPPH scavenging capacity, followed by colon, appendix, pancreas, and rectum. Liver and lung displayed the weakest DPPH scavenging capacity. These values are similar to those reported by Di Bernardini et al. (2012) for papain-hydrolyzed bovine brisket muscle. However, only 1 mg/mL was used in that study compared with 5 mg/mL in ours. In general, our values for DPPH radical scavenging are low compared to other studies. Alcalase-hydrolyzed porcine liver (3 mg/mL) was found to exhibit ∼40% DPPH radical scavenging capacity after 3-h hydrolysis (Yu et al. 2012). Myofibrillar protein hydrolysates were reported to display DPPH radical scavenging capacities of ∼65% and ∼70% after 24 h of actinase E or papain treatment, respectively (Saiga et al. 2003). However, no protein concentration was specified in the latter study, making comparisons difficult. The hydrolysate concentration has been shown to have a dose-dependent effect on the DPPH radical scavenging activities of tannery fleshings (Balakrishnan et al. 2011), underlining the importance of reporting concentrations for scavenging capacities. However, as recently pointed out, the possibility of comparing DPPH antioxidant capacities between laboratories is complicated by the wide variation in methods which result in highly variable values, even with well-known standards (Sharma and Bhat 2009). Therefore, comparisons should be made with caution, which also applies to other methods, for example, iron chelation and ABTS radical scavenging.

##### ABTS versus DPPH

To compare the usability of the ABTS and DPPH radical scavenging assays for our particular samples, both methods were employed. Since both assays are based on electron transfer mechanisms involving the reduction in colored prooxidants, they would be expected to yield similar results. On the other hand, since the DPPH assay is performed in an organic solvent system, it is more suited to lipophilic compounds or compounds with a high lipid content, whereas this is not the case for the ABTS assay, which is compatible with both aqueous and organic solvent systems (Arnao et al. 2001). Other studies have compared the two assays, and the antioxidant capacity detected by the ABTS assay has been reported to be significantly higher for a variety of different foods compared to that of the DPPH assay, partially because the highly pigmented and hydrophilic antioxidants are better reflected by the ABTS assay than the DPPH assay (Kim et al. 2002; Floegel et al. 2011), suggesting that the ABTS assay may be better than the DPPH assay for detecting antioxidant capacity in a range of different foods. Also, in this particular study the values from the ABTS assay were higher than those from the DPPH assay, showing the higher sensitivity of the former assay, which is in agreement with other studies (Sachindra and Bhaskar 2008; Foh et al. 2010; Balakrishnan et al. 2011). Nevertheless, the order of hydrolysates, ranging from high to low radical scavenging capacity, was the same for both assays, except for the lung and heart samples. Interestingly, the ABTS assay placed lung as having the highest capacity and heart as having the lowest, which was the exact opposite of the DPPH assay. We have no good explanation for this.

##### Tissue and mechanism

None of the hydrolysates displayed a superior capacity when tested in all four assays. Instead, a relatively wide distribution of performance across assays and hydrolysates was observed. Liver, colon, and appendix displayed the highest values of inhibition of lipid oxidation as well as the first, second and fourth highest value in the iron-chelating assay, respectively. This suggests an antioxidant mechanism of these hydrolysates, namely that they bind the hemin which is used to initiate the oxidation, thereby impairing it. The high iron chelation capacity of the liver hydrolysate suggests that it contained antioxidant peptides mainly working as iron chelators or contained a higher concentration of heme pigments which could chelate iron. The colon and appendix hydrolysates also displayed high antioxidative capacities as scavengers, indicating that these hydrolysates contain peptides that can operate as scavengers in addition to peptides acting as iron chelators. We assume that the majority of the hydrolysates consist of peptides, and that they are responsible for the antioxidant capacity, although, the hydrolysates may contain endogenous compounds, for example, ascorbic acid, which can contribute to the overall antioxidant capacities. However, we aimed to investigate the antioxidant capacities of the hydrolyzed tissues as a whole, that is including potential endogenous antioxidant or oxidizing compounds (e.g., iron).

Table 3 shows the order of the hydrolysates arranged from highest to lowest capacity within each assay. The different antioxidant mechanisms displayed by the various tissues also point to the advantage of mixing hydrolysates, as it would inhibit a broader range of oxidative processes.

**Table 3:** Schematic representation of the antioxidant capacity of the hydrolysates ranked from highest to lowest capacity

	Lipid oxidation inhibition	Iron chelation	ABTS	DPPH
Highest antioxidative capacity	Colon^ab^	Liver^a^	Lung^a^	Heart^a^
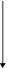	Liver^a^	Colon^b^	Colon^a^	Colon^b^
	Appendix^a^	Pancreas^b^	Appendix^ab^	Appendix^b^
	Lung^ac^	Appendix^b^	Pancreas^ab^	Pancreas^c^
	Pancreas^ac^	Rectum^c^	Rectum^ab^	Rectum^d^
	Heart^c^	Lung^d^	Liver^ab^	Liver^e^
Lowest antioxidant activity	Rectum^c^	Heart^e^	Heart^b^	Lung^e^

Hydrolysates with different letters in the same columns are significantly different at *P* < 0.05. ABTS, 2,2′-azino-bis (3-ethylbenzothiazoline-6-sulfonic acid); DPPH, 2,2-Diphenyl-1-(2,4,6-trinitrophenyl) hydrazyl.

## Conclusion

All tissues showed antioxidant capacity upon hydrolysis with Alcalase and Protamex. Hydrolysates from colon, liver, and appendix were particularly efficient at inhibiting lipid oxidation, possibly due to their iron-chelating properties. Furthermore, colon and appendix hydrolysates also displayed high capacities for radical scavenging, indicating a broad antioxidant potential. Our results show that animal by-products can be transformed into antioxidant hydrolysates, potentially creating added value. The applicability of these hydrolysates as antioxidants in real food matrices remains to be determined.
